# Fire, climate change and biodiversity in Amazonia: a Late-Holocene perspective

**DOI:** 10.1098/rstb.2007.0014

**Published:** 2008-02-11

**Authors:** M.B Bush, M.R Silman, C McMichael, S Saatchi

**Affiliations:** 1 Department of Biological Sciences, Florida Institute of Technology150 West University Boulevard, Melbourne, FL 32901, USA; 2 Department of Biology, Wake Forest UniversityBox 7325 Reynolda Station, Winston Salem, NC 27104, USA; 3 Jet Propulsion Laboratory, California Institute of Technology4800 Oak Grove Drive, Pasadena, CA 91109, USA

**Keywords:** neotropical, charcoal, fire, migration, solar minimum, warming

## Abstract

Fire is an important and arguably unnatural component of many wet Amazonian and Andean forest systems. Soil charcoal has been used to infer widespread human use of landscapes prior to European Conquest. An analysis of Amazonian soil carbon records reveals that the records have distinct spatial and temporal patterns, suggesting that either fires were only set in moderately seasonal areas of Amazonia or that strongly seasonal and aseasonal areas are undersampled. Synthesizing data from 300 charcoal records, an age–frequency diagram reveals peaks of fire apparently coinciding with some periods of very strong El Niño activity. However, the El Niño record does not always provide an accurate prediction of fire timing, and a better match is found in the record of insolation minima. After the time of European contact, fires became much scarcer within Amazonia. In both the Amazonia and the Andes, modern fire pattern is strongly allied to human activity. On the flank of the Andes, forests that have never burned are being eroded by fire spreading downslope from grasslands. Species of these same forests are being forced to migrate upslope due to warming and will encounter a firm artificial fire boundary of human activity.

## 1. Introduction

Climate change threatens Amazonian biodiversity both directly and indirectly (e.g. Nepstad *et al.* 2004). The only climate model that captures feedbacks among atmosphere, ocean and vegetation predicts a near-permanent El Niño-like state in the Pacific and growing drought across Amazonia this century (Cox *et al.* 2004). Using the IS92a ‘business-as-usual’ scenario, this model predicts that almost all of Brazilian Amazonia will be too dry to support rainforest by AD 2100 (Shindell *et al.* 2001; Cox *et al.* 2004). Other climate models make less dire predictions, but almost all project a significant loss of forest cover due to climate change this century.

While climate change can induce forest dieback on century time scales, fire is a much more immediate threat (Barlow & Peres 2004; Nepstad *et al.* 2004). Based on the exponential increase in the area burned in Amazonia over the last 30 years, fire associated with human activity and drying is likely to be what eliminates the forest rather than the gradual stress of climate change (Nepstad *et al.* 2004).

Understanding the fire ecology of Amazonian systems is critical to establishing conservation policy, and also to understand the current vegetation dynamics, carbon balance and successional state (Barlow & Peres 2004). One key tool in developing such an understanding is to use the pre-historic record to assess the effects of fire during past phases of El Niño activity and periods of human disturbance. In this paper, we highlight examples of past and projected interactions of climate, fire and human activity.

### (a) Pre-Columbian fires in Amazonia

Forest fire in much of Amazonia is a synergistic consequence of drought and human activity. The escape of human-set fires to become wildfires under El Niño-induced drought conditions in Kalimantan in 1997–1998 (van Nieuwstadt *et al.* 2001), and Amazonia in the droughts of 1997–1998 and 2002–2005 (Marengo *et al.* submitted) are examples of such interactions. Also, especially in evergreen forests, fires are more easily started during drier versus wetter periods, linking overall precipitation levels and human activity (e.g. Cochrane *et al*. 2002).

Not all of Amazonia is equally likely to burn. Nepstad *et al.* (2004) identified seasonality, drought duration and soil type as key factors in determining the flammability of Amazonian systems. Almost all contemporary Amazonian fires are either set deliberately, accidental or intentional burns that escape to become a wildfire (e.g. Hammond & ter Steege 1998). In mesic Amazonian systems, truly natural fires are very rare (Cochrane *et al.* 2002), and some palaeoecological records spanning thousands of years show no indication of fire (e.g. Bush *et al.* 2007*a*,*b*). The prevailing view is that natural fire in Amazonian rainforest is so rare that charcoal is interpreted to indicate human activity. During known dry times, such as the Mid-Holocene drought episodes between *ca* 8000 and 5000 cal. yr BP, it is plausible that natural fires occurred in most seasonal settings (see Mayle & Power 2008), and may have contributed to the large-scale patterns of plant species diversity found in the Amazon basin (Silman 2007).

Whatever the source of ignition, fire occurrence is related to drought in Amazonia. Such droughts can be induced by weather patterns in both the Pacific and Atlantic Oceans, and El Niño *et al*. events have been linked to some of the strongest periods of fire activity in the last decades (Kitzberger *et al.* 2001). However, unusual warmth in the northern tropical Atlantic also induces droughts and may be linked to fire activity (Marengo 2007). The warm water sets up a see-saw oscillation in the Hadley cells that displaces the intertropical convergence zone northwards, leading to drought in northern Amazonia (Baker 2002). Such an influence of this Atlantic oscillation is the strongest when El Niño Southern Oscillation (ENSO) is relatively weak (Marengo *et al.* submitted), meaning that there can be drought independent of ENSO. The generally opposite phasing of precipitation in the northern tropical Atlantic and Amazonia may be influenced by variability in solar output (Hodell *et al.* 2001; Shindell *et al.* 2001). Periods of high solar output coincide with droughts in the Caribbean and floods in Amazonia (e.g. Mauas & Flamenco 2005), while low solar output has the opposite effect.

Under normal conditions of moisture, the probability of fire decreases exponentially with distance from roads and clearings (Cochrane & Laurance 2002), demonstrating that the initiation of these fires is largely a result of human activity. In a study of forests near Tailândia, Brazil, Cochrane & Laurance (2002) found a subdecadal return period for fire at the edge of forest fragments, whereas at a distance of more than 2500 m into the forest the fire frequency was the same as background level (i.e. no fires documented in the last 500–1000 years). These data indicate that in these moderately seasonal forests, fires almost always burn themselves out within a few hundred metres and do not spread to form a large wildfire. However, during droughts, fires initiated by human activity can spread as wildfires. In the Brazilian state of Acre, at the epicentre of the 2005 drought, the area of leakage forest fires was more than five times greater than the area directly deforested. Fire leakage into flammable forests may be, therefore, the major agent of biome transformation in the event of increasing drought frequency (Aragão *et al.* 2007).

In summary, fire frequency is some function of both climate and human interventions. The vast majority of Amazonian fires are sparked by humans and fire spreads most readily in areas and times of high flammability. These observations lead to basic testable predictions for the palaeoecological record: (i) Amazonian fires of the past are primarily associated with human disturbance, (ii) the highest proportion of sites exhibiting fire will coincide with the driest periods of Amazonian history (rather than the time of the highest population) and that (iii) highly seasonal settings will exhibit a higher incidence of fire than aseasonal ones.

### (b) The palaeoecological record

Charcoal analysis has become a standard tool used to assess past fire histories (e.g. Horn & Sanford 1992; Whitlock & Larsen 2001; Mayle *et al.* 2007). Soil charcoal has also been widely used to date past fire events via ^14^C (e.g. Hammond *et al.* 2006; Titiz & Sanford 2007) and, while there may be uncertainties for some forest types (Gavin 2001), dating is expected to be within the resolution of ^14^C dating errors.

**Figure 1:**
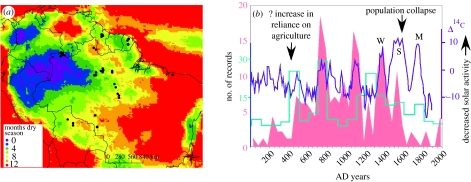
The pre-Columbian peak of Amazonian soil charcoal. (*a*) Map of seasonality (number of months with more than 100 mm of rain) as determined from tropical rainfall monitoring mission (TRMM; Silman 2007), showing locations of soil charcoal ^14^C dated samples. (*b*) Two hundred and twenty-eight ^14^C ages for dated charcoal horizons in Amazonian soils (see Bush & Silman 2007 for review). All ages were calibrated using Calib v. 5.0.2 (Stuiver & Reimer 1993), and midpoint ages per 50-year period plotted against time (pink). Purple indicates atmospheric Δ^14^C as a proxy for solar output (Bard 1998). M, maunder; S, sporer; W, wolf solar minima. Turquoise indicates number of El Niño events per century (Moy *et al.* 2002).‘?’ indicates uncertainty as to the timing of a shift in Amazonian cultural adaptations.

The majority of sites where soil charcoal has been documented in Amazonia lie in moderately seasonal forests (figure 1*a*). While extreme drought may terminate with a lightning event that sparks a fire, it is far more probable that humans initiate the fires (Bush *et al.* 2007*a*). The synergy between human activity and drought comes when flammability increases; small fires initiated by humans can run out of control and become large wildfires.

One possibility is that the observed pattern is a product of where modern infrastructure and accessibility has focused sampling (*sensu* Nelson *et al*. 1990). An alternative (not mutually exclusive) explanation is that the wettest areas of western Amazonia have very low fire frequencies, with very little chance of wildfire escape. Indeed, the burned sites in the wetter forests of San Carlos near the Venezuelan border were directly associated with potsherds (Sanford *et al.* 1985; Saldarriaga & West 1986) indicating local human presence. As seasonality increases, the risk of wildfire through fire escape increases. Even the Guyanan forests sampled by Hammond *et al.* (2006), although not particularly seasonal, have some of the highest interannual variability in rainfall and are very drought prone (Hammond *et al.* 2006). The driest areas of Amazonia have yet to provide soil charcoal records. Given the known use and occupation of the savannahs (e.g. Erickson 2001), it is considered probable that soil charcoal will be found in those locations as surveys are expanded.

Temporally, the pattern of fire events does not coincide with a simple trajectory of increasing human population and settlement prior to a crash in the 1500s–1600s. A surge in fire events is evident between *ca* AD 200 and AD 600, and this coincides with archaeological data for an increased adoption of agriculture that reaches a peak with the formation of *terra preta* soils (Amazonian dark earths) between AD 400 and AD 800 (Neves *et al.* 2004; figure 1*b*). The peak of fire frequency at *ca* AD 800 coincides with a peak of inferred El Niño activity (Moy *et al.* 2002). At *ca* AD 700–800 and AD 1000–1100, drought is inferred to have promoted fire escape; El Niño-induced drought may have spurred wildfires. However, the El Niño pattern alone is not enough to lead to accurate predictions of past fire activity. For example, another active El Niño period in the 1300s has no corresponding peak of Amazonian fires.

Schimmelmann *et al.* (2003) suggested an approximately 200-year periodicity to flood and drought regimes in Amazonia and attributed the pattern to a blend of El Niño Southern Oscillation variability and solar activity. Indeed, modern empirical data from Amazonia show a strong positive relationship between high solar radiance and peak discharge of the Parana River (Mauas & Flamenco 2005). Again, recognizing the limitations of this dataset, the coincidence of peaks of charcoal occurrence with minima of solar output and general trends of increased solar output matching few charcoal records are highly suggestive that solar input has played a substantial role in establishing the pre-Columbian pattern of fires (figure 1*b*). The translation of solar minima into climatic change is unclear, with suggested mechanisms being weakening the thermohaline circulation (Bond *et al.* 2001) or via reduced cloud formation as a result of interactions with cosmic rays (Svensmark & Friis-Christensen 1997).

The insolation minima are associated with Amazonian drought (above), but post-1600 insolation minima did not engender a fire signature because human populations were collapsing and agriculture was abandoned (Myers 1988; Black 1992; Denevan 2003). That these strong insolation minima did not result in large peaks of fire activity is further evidence of the importance of humans as initiators of fire. We hypothesize that the majority of fire records between AD 200 and AD 1400 resulted from fire escape rather than necessarily reflecting intentional human manipulation at that exact site.

**Figure 2:**
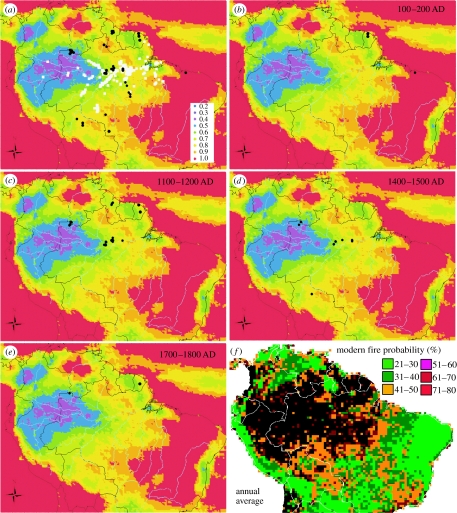
Spatial and temporal patterns of known fire sites at selected time slices mapped with climate variability. (*a*) All records, (*b*) AD 100–200, (*c*) AD 1100–1200, (*d*) AD 1400–1500 and (*e*) AD 1700–1800. Charcoal data are the same as in figure 1 legend. Climate variability of monthly means of precipitation are based on TRMM data 1998–2004 (warmer colours are more variable; Silman 2007). Light grey spots are locations of known *terra preta*. Black spots represent locations of fires dated to within the time slice. (*f*) Modern probability of fire (%) occurrence for any given year averaged over 17 years derived from Advanced Very High Resolution Radiometer (AVHRR) satellite observation.

Another way to visualize the pattern of fires is to look at the spatial distribution of known fires within given time slices (figure 2*a*–*f*). The time slices are selected to represent times of high and low fire frequency, and the period before and after European Conquest. *Terra preta*, a strong indicator both of human presence and some localized fire activity, is present along many of the major Amazon channels, especially in moderately seasonal areas. In the soil charcoal data, there is some, but not a complete, overlap with the *terra preta*, possibly suggesting different styles of management or that some areas were more prone to accidental fires than others. Although the northern half of the Amazon basin responds differently to El Niño forcing than the southern half (Marengo 2007), no clear north–south or east–west oscillation is apparent in the charcoal data time slices. From these limited data, it appears that fires were either present across a relatively wide area or they were similarly absent.

**Figure 3:**
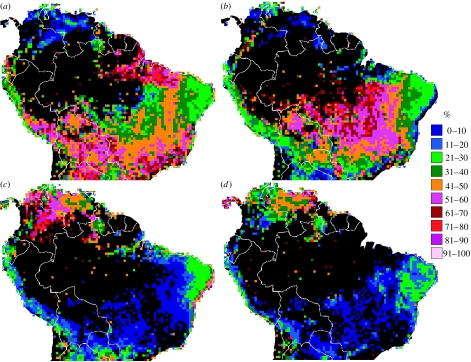
Seasonal probabilities of fire occurrence in Amazonia. (*a*) September to November, (*b*) June to August, (*c*) December to February and (*d*) March to May. Colours represent the cumulative probability of fire occurrence for any season over 17 years derived from AVHRR satellite observation. Percentages represent the probability of an area burning in a given year. The seasonality of fires closely follows seasonality in rainfall.

The contemporary fire history observed by satellite data (figure 3*a*–*d*) shows a clear distribution of fire probability related to both the climate and human-induced fragmentations across the Amazon basin. The probability of fire occurrence in a given year and averaged over 17 years (1982–1999) shows two patterns: (i) areas with high rainfall and short-to-moderate dry seasons in the central and western Amazon basin have very low probability of fire occurrence and (ii) areas along the arc of deforestation and longer dry seasons have high probability of fire (Carmona-Moreno *et al.* 2005). An important aspect of defining the fire regime in Amazonia is the probability of fire occurring in a particular season for a given area (figure 3). The cumulative probability of seasonal distribution of fire derived from the satellite observation over the same period (1982–1999) indicates low probability of fire occurring in the first two quarters of the year associated with the rainy seasons December–January–February and March–April–May. Fire seasons are stable over the entire 17-year record, suggesting a high concentration of fire during dry seasons June to August and September to November with approximately three trimesters shift between fire activity in the Northern and Southern Hemisphere in Amazonia. One can hypothesize that although interannual variations in climate (e.g. ENSO events) may impact the intensity of fire occurrence, they have little or no effects on the fire seasonality.

The full geographical extent of fires associated with insolation minima is not known. If these were local fires that failed to spread, then biodiversity impacts would have been minimal. At the other extreme, if all the seasonal forests of Amazonia were burning, there would have been significant impacts on both biodiversity and climate, i.e. contributing to the medieval warm period (*sensu* Ruddiman 2003). Species that benefit from the early successional forests, which anthropogenic activity generates, and human commensals would have increased in abundance, while those that are obligate deep forest inhabitants would have faced local extinction (Pearsall 2002; Barlow *et al.* 2003; Barlow & Peres 2004).

The ongoing climate changes are projected to reduce precipitation over much of Amazonia, with the eastern half of the basin supporting savannah rather than forest within the next 50–100 years. As forests generate their own microclimate, they can survive at lower precipitation than the amount needed for them to regenerate (Sternberg 2001). While this observation may indicate that climate models are likely to exaggerate the loss of forest, the encroachment of fire as a result of human activity would more than offset that microclimatic buffer (see Barlow & Peres 2008). The net result of climate change and human encroachment is likely to be one of large-scale forest loss with fire rather than climate change being the immediate agent of destruction (Cochrane *et al.* 2002; Nepstad *et al.* 2004).

## 2. Climate change and fire history on the flank of the Andes: a Peruvian case study

The second example that we wish to discuss is the interaction between fire, climate change and human activity on the Andean flank. The foothills and eastern slope of the Andes harbour exceptional biodiversity. Very few data on fire histories exist for these forests. To date, palaeoecological analyses of records from lake sediments within modern cloud forest settings have not yielded charcoal (H. Hooghiemstra 2007, personal communication), indicating the absence of human occupation and natural fires. Near the upper elevational limit of woodland growth, local charcoal concentrations increased markedly at *ca* 11 700 cal. yr BP in records from Peru and Bolivia: Lakes Titicaca (16° S, 3800 m), Caserococha (13° S, 3900 m; Paduano 2001) and Chochos (6° S, 3300 m; Bush *et al.* 2005). Fires are thought to have reduced woodland cover, especially *Polylepis*, the fire-sensitive genus of treelet that, in the absence of fire, forms the highest woodlands in the Andes (Kessler 1995).

Within the last 3300 years, the overall climatic trajectory on the Andean flank has been towards wetter conditions (Abbott *et al.* 2003; Bush *et al.* 2005), though there have been distinct drought events inferred within that time (Meggers 1994; Abbott *et al.* 2003). On first principles, a wet period would be expected to limit fire frequency and allow some upslope invasion of trees into the puna. Such periods can promote fuel build-up, allowing larger and hotter fires to burn during the next fire cycle (Kitzberger *et al.* 2001). Treeline has probably been artificially depressed during this period by human-induced burning for pastoralism and cropping (Ellenberg 1958). Consequently, the modern treeline that commonly occurs between 3200 and 3500 m elevation in Peru would, in the absence of human activity, lie closer to 3700–3900 for many species, with *Polylepis* perhaps forming open woodlands up to 4800 m elevation (Ellenberg 1958; Kessler 1995).

Fire-generated limits to altitudinal migration are likely to be a major threat to Amazonian diversity for species whose ranges lie above 2600 m in the Andes. Ongoing climate change is expected to induce upslope migrations of plants and animals to maintain their bioclimatic envelopes (Malcolm *et al*. 2006). The Hadley CM3 model suggests an approximately 3–5°C warming for the Peruvian Andes by the end of this century (Cox *et al.* 2000). Moist air adiabatic lapse rates are approximately 5°C in this section of the Andes, and thus a vertical migration of approximately 600–1000 m would be required to maintain an equivalent temperature. While migration distances are very short, normally 20–40 km laterally to achieve that gain in altitude, significant barriers to dispersal may still exist. As people continue to clear the upper limit of the cloud forest and fire penetrates lower, there will be no opportunity for species to migrate upwards across this fire line. We term this upward pressure from climate change met by the downward pressure of land use the ‘Big Squeeze’.

At the foot of the Andes and on the lower Andean flank, fire will be a very significant threat. Although relatively wet in many areas, this region has been extensively exploited for coffee, coca, rice and other crops (Bush 2002). In any area where there is ready access, little natural habitat remains between 500 and 1500 m elevation, forming a green gap between large areas of relatively intact forest above and below this range. With improved communication and transportation, this cultivable section of the Andes will be even more heavily exploited. If, as some models predict, cloud base lifts in response to warmer conditions, the green gap will press upwards. Pressing down from above will be human land use.

Given the residence time of greenhouse gases, and increasing industrialization, it would be unduly optimistic to expect no need for climate-forced migration over the next century. Averting the worst effects of the ‘Green Gap’ and the ‘Big Squeeze’ will require vertical migration corridors to be established. Taking degraded lands into conservation now, so that succession can start, may mean that in 50–100 years time, they will be part of the ecological conduit rather than a barrier. Changing land management, especially burning practices near treeline, and substituting low-density grazing land with carbon sequestering forests may provide an alternate source of revenue and be of immense ecological value.

## 3. Conclusions

Fire histories for Amazonia are only just beginning to become available. A close correlation between human activity and burning is found, while under other circumstances fire is generally very rare. Pre-Columbian occupants of Amazonia burned the forest to clear it for agriculture, and perhaps also to improve hunting. Soil charcoal data suggest that episodes of wildfire coincide with insolation minima as droughts beset Amazonia. The spatial extent of these fires has yet to be resolved, and thus their impact on regional biodiversity cannot be estimated. However, from the preliminary data, it appears probable that the impacts of fire were rather local, and do not correspond to the regional conflagration associated with the advancing front of deforestation observed in recent times.

In the Amazon lowlands, increasing flammability due to the synergy of projected climate change and forest fragmentation strongly suggests that by 2050 much of eastern Amazonia will be fire prone. A few species will be well suited to the new conditions (mainly those that also live outside mesic forest settings), but for many more, usable habitat will be lost and local, perhaps global, extinction will follow. In western Amazonia, the wetter conditions will probably limit the spread of fire, but increased penetration of currently intact forests for mineral extraction, transportation or cultivation will greatly enhance the probability of wildfire. Even where fire does not form a threat, increasing human populations accessing bushmeat may set in motion trophic cascades that have largely unforeseen consequences (Redford 1992; Silman *et al.* 2003; Terborgh *et al.* 2006).

On the eastern flank of the Andes, the cloud forest, an unparalleled system for biodiversity, will be pressured from forced upslope migration in response to climate change and the downslope erosion of treeline as a result of burning Andean pastures. Conservation measures are needed now to minimize the effects of climate change and to promote sustainable land use that will facilitate climate-induced species migration.
